# Effects of the polyunsaturated fatty acids, EPA and DHA, on hematological malignancies: a systematic review

**DOI:** 10.18632/oncotarget.24405

**Published:** 2018-02-05

**Authors:** Milad Moloudizargari, Esmaeil Mortaz, Mohammad Hossein Asghari, Ian M. Adcock, Frank A. Redegeld, Johan Garssen

**Affiliations:** ^1^ Department of Immunology, School of Medicine, Student Research Committee, Shahid Beheshti University of Medical Sciences, Tehran, Iran; ^2^ Department of Immunology, School of Medicine, Shahid Beheshti University of Medical Sciences, Tehran, Iran; ^3^ Clinical Tuberculosis and Epidemiology Research Center, National Research Institute for Tuberculosis and Lung Disease (NRITLD), Shahid Beheshti University of Medical Sciences, Tehran, Iran; ^4^ Division of Pharmacology, Utrecht Institute for Pharmaceutical Sciences, Faculty of Science, Utrecht University, Utrecht, Netherlands; ^5^ Department of Pharmacology, Faculty of Medicine, Babol University of Medical Sciences, Babol, Iran; ^6^ Cell and Molecular Biology Group, Airways Disease Section, National Heart and Lung Institute, Imperial College London, Dovehouse Street, London, UK; ^7^ Nutricia Research Centre for Specialized Nutrition, Utrecht, Netherlands

**Keywords:** omega-3, eicosapentaenoic acid, docosahexaenoic acid, apoptosis, fish oil

## Abstract

Omega-3 polyunsaturated fatty acids (PUFAs) have well established anti-cancer properties. Eicosapentaenoic acid (EPA) and docosahexaenoic acid (DHA) are among this biologically active family of macromolecules for which various anti-cancer effects have been explained. These PUFAs have a high safety profile and can induce apoptosis and inhibit growth of cancer cells both *in vitro* and *in vivo*, following a partially selective manner. They also increase the efficacy of chemotherapeutic agents by increasing the sensitivity of different cell lines to specific anti-neoplastic drugs. Various mechanisms have been proposed for the anti-cancer effects of these omega-3 PUFAs; however, the exact mechanisms still remain unknown. While numerous studies have investigated the effects of DHA and EPA on solid tumors and the responsible mechanisms, there is no consensus regarding the effects and mechanisms of action of these two FAs in hematological malignancies. Here, we performed a systematic review of the beneficial effects of EPA and DHA on hematological cell lines as well as the findings of related *in vivo* studies and clinical trials. We summarize the key underlying mechanisms and the therapeutic potential of these PUFAs in the treatment of hematological cancers. Differential expression of apoptosis-regulating genes and Glutathione peroxidase 4 (Gp-x4), varying abilities of different cancerous and healthy cells to metabolize EPA into its more active metabolites and to uptake PUFAS are among the major factors that determine the sensitivity of cells to DHA and EPA. Considering the abundance of data on the safety of these FAs and their proven anti-cancer effects in hematological cell lines and the lack of related human studies, further research is warranted to find ways of exploiting the anticancer effects of DHA and EPA in clinical settings both in isolation and in combination with other therapeutic regimens.

## INTRODUCTION

Omega 3 polyunsaturated fatty acids (PUFAs) are members of a large group of fatty acids (FAs) possessing multiple double bonds in their structures [1]. These biologically active macromolecules are well known to have various vital physiological roles required for proper functioning of cells [1]. Many effects of these FAs are mediated by their more active longer chain metabolites which act via changing the plasma membrane composition, producing various inflammatory mediators and altering the expression of different genes. Eicosapentaenoic acid (EPA) and docosahexaenoic acid (DHA) are among the very long chain PUFAs for which various health benefits have been shown. These FAs decrease the risk of cardiovascular diseases by regulating the contributing risk factors including blood pressure, blood coagulation, cardiac rhythm and heart rate among many others [2–4]. Anti-inflammatory [5] and immune modulatory effects [1, 6] and the regulation of several metabolic pathways by these FAs have been also reported giving them the potential to be therapeutically exploited in the treatment of inflammatory diseases, metabolic disorders such as type-2 diabetes and immunocompromising conditions.

An epidemiological study in 1997 revealed that decreased incidence of colorectal cancer among South African West Coast fishermen who generally had a less healthy diet in comparison with the urban population, could be attributed to presence of fish oil in their diet [7]. Dietary intake of omega-3 FAs has been also shown in human studies to inversely correlate with the overall risk of different types of malignancies such as colorectal, prostate and breast cancer [8–10]. A substantial number of *in vitro* studies on cancer cell lines as well as studies on animal models of cancers have shown the anti-proliferative, apoptotic, cytotoxic, and anti-metastatic properties of DHA and EPA [11]. Keeping in mind that ROS can decrease cancer cell survival [12], different mechanisms have been suggested for the anti-cancer effects of DHA and EPA such as induction of ROS and consequent peroxidation of lipids [13, 14], changing the composition of the plasma membrane and lipid rafts [15, 16], affecting the mitochondrial membrane potential [17] and epigenetic alteration of genes involved in apoptosis [18].

Potential drug sensitizing effects of DHA and EPA have also been reported in numerous studies such that low amounts of these two FAs in combination with anticancer agents can result in increased sensitivity of cancer cells to anti-neoplastic agents even in some drug-resistant cell lines [19]. Recent evidence also points at the potent and at the same time selective actions of EPA and DHA on multiple myeloma cell lines which had not been previously investigated [20]. Most of the well-established anti-cancer effects of these PUFAs have been studied in solid tumors. Although ample data is available regarding the effects of DHA and EPA on haematological malignancies, still there is ambiguity regarding the exact mechanisms responsible for their actions on haematological cancers. In the present study, we systematically reviewed the effects of DHA and EPA on different leukemic and multiple myeloma cells with particular focus on the potential mechanisms of action. Moreover, we review the current evidence on the bioavailability and applicability of EPA and DHA for their clinical use in the context of haematological cancers.

### Search strategy and data extraction

**Figure 1:**
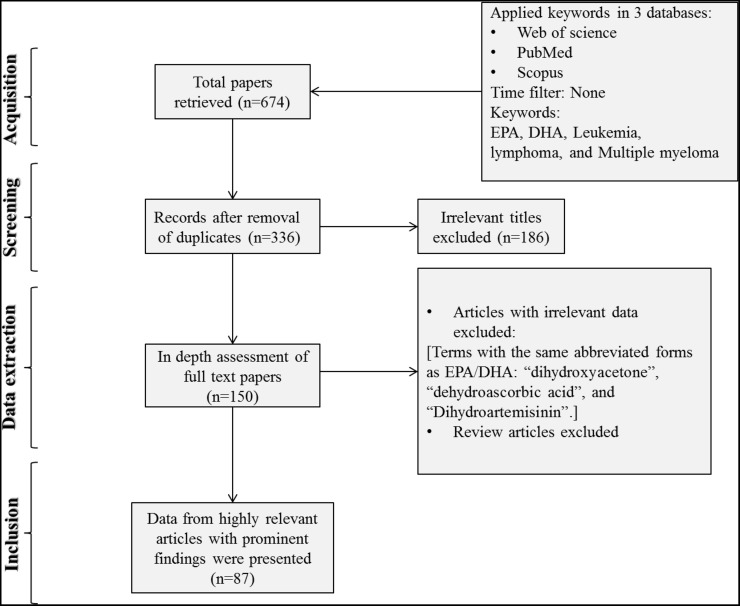
Flow diagram of the search strategy “Leukemia” AND “DHA OR EPA” and “Multiple Myeloma” AND “DHA OR EPA” were searched in three databases of Web of Science, Pubmed, and Scopus. Finally 133 papers were deemed eligible to be reviewed.

In order to access the relevant data, a literature search was performed based on the Preferred Reporting Items for Systematic Reviews and Meta-Analyses (PRISMA) guidelines. The authors explored the Web of Science, Pubmed, and Scopus databases using the following keywords: “Leukemia” AND “DHA OR EPA”, “Multiple Myeloma” AND “DHA OR EPA”, and “Lymphoma” AND “DHA OR EPA”. EPA and DHA are abbreviations which are frequently used to show eicosapentaenoic and docosahexaenoic acids, respectively. In total, 674 published papers were retrieved after applying the filter of “articles in English only”. After removing the duplicates, the articles were screened based on their relevance to the topic and all irrelevant papers were excluded. The studies where the term “lymphoma” was detected in the context of the extended form of bcl-2 (B cell lymphoma 2) and were found irrelevant to the topic were also removed. The full texts of the remaining papers (n=150) were further evaluated for the eligibility and relevance of their findings. All discrepancies were subjected to discussion until proper conclusions were made in each case. A final number of 87 articles met all the inclusion criteria and were found suitable to be reviewed (Figure 1). Data extraction was performed and the key findings of all previous studies were presented as tables and illustrations (Table 1 and Figure 1). The results were organized in separate sections including *in vitro* and *in vivo* studies and drug sensitizing effects. Finally, the overall results were subjected to discussion in which the possible mechanisms of selective action of EPA and DHA on neoplastic cells and the feasibility of their clinical usage were explained and a conclusion was finally drawn.

**Table 1:** The effects of EPA and DHA treatment on different cell lines and the suggested mechanisms

Cell line animal model	Specification	EPA	DHA	Most effective dosage(s)	Incubation time(s)	Outcomes	Reference
**U 266**	B lymphocyte (lymphoblast)	*	*	50 μM 100 μM		Apoptosis induction, Drug sensitizing (Bortezomib), No effect on normal PBMCs Caspase 3 activation, Hypoxia modulation, NF-kB inhibition, Mitochondrial perturbation (↑mitochondrial membrane potential (ΔΨm)), Upregulation of genes involved in oxidative stress	[20]
**L363**	plasma cell leukemia	*	*				
**OMP-1/2**	multiple myeloma	*	*				
**U937**	Monocytic leukemia		*	10 μM 20 μM		Apoptosis induction, Caspase 3 activation and ROS generation as a result of: ↑[Ca2]_i_ via PLC/IP_3_ pathway and PKCγ/δ activation	[47]
**Jurkat**	Human T cells	*	*	10 μM 20 μM		↑[PH]_I_, simple diffusion, (flip-flop) of the fatty acid, occurring in phospholipid bilayers of the plasma membrane (the long-lasting acidification was dependent on increases in [Ca2]_i_)	[48]
**Molt-4**	Leukemic cell line	*	*	5-10-20 μg		Antiproliferative/cytotoxic effect in both normal and leukemic lymphocytes (concentration dependent) ↑IL-2 (in leukemic cells), ↓IL-2 (in normal cells), Free radical generation (↑SO_2_^−^ and lipid peroxidation (↑MDA)	[84]
**HL-60**	Human promyelocytic leukemia cells	*	*	60 μM	6, 12 h	Growth inhibition and apoptosis induction, ROS generation, Activation of caspase-like proteases (3, 6, 8, and 9-not 1), Activation of Bid cleavage, Decreasing mitochondrial membrane potential, Cytochrome c release from mitochondria, Surfactant effect on membrane	[17]
**HL-60**	Human promyelocytic leukemia cells	*	*	As parts of lipid preparations	NM	Antiproliferative effect NM	[23]
**U937**	promonocytic cell line	*		100 μM	1, 3, and 24 h	Regulating gene expression ↑monocytic (leukemia) cell differentiation, Proliferation inhibition, ↑ demethylation of a specific CpG site in interon 1 on H-Ras gene, ↑H-Ras isoform mRNA and protein expression ↑active phosphorylated forms of C/EBPβ and ERK1/2, ↑proteins expression of CCAAT/enhancer-binding proteins (C/EBP and C/EBP), PU.1, and c-Jun (myeloid lineage-specific transcription factors)→ ↑expression of M-CSF recepror	[18, 46]
**HL-60 and K-562**	Human acute promyelocytic leukemia HL-60 and chronic myelogenous leukemia K-562 cell lines, respectively	*		NM	24 h	Changes in cell cycle: ↑G0/G1, ↓G2/M ↑Necrosis ↑Apoptosis (only in HL-60) ↑bcl-2 expression	[36]
**HL-60 and K-562**	Human acute promyelocytic leukemia HL-60 and chronic myelogenous leukemia K-562 cell lines, respectively	*		40, 60 and 120 μM	24 and 72 h	↑Necrosis ↑Apoptosis (only in HL-60) Independent of DNA fragmentation	[38]
**HL-60**	Human acute promyelocytic leukemia		*	10-160 μM	24, 48 and 72 h	↑Apoptosis ↓Proliferation Phosphorylation-dependent inactivation of Rb protein Inducing cell cycle arrest at G0/G1 phase ↑Expression of E2F-6 and Bax	[41]
**CEM, HL-60, MM1.R, and MM1.S**	T-cell leukemia, myeloid leukemia, myeloma, and myeloma, respectively		*	100 μM	16 h	↑Sensitivity to DHA cytotoxic effects ↓GP-x4 expression	[66]
**Raji**	human B-cell lymphoblastoid line		*	100 μM	72 h	Synergizes the effect of clioquinol with regard to the following parameters: ↑Apoptosis ↓Cell viability ↓NF-κB activity ↓Levels of Akt, p-65 and Bcl-2	[68]
**EHEB, MEC-2** **JVM-2**	B-CLL-derived cell lines B-PLL-derived cell line	*	*	55, 75, and 100 μM	72 h	↓Cell viability ↑Lipid peroxidation ↑ROS production Cell cycle arrest in G2/M	[85]
**C57/BL6 mouse**	Receiving transplantation of HSCs expressing Bcr-Abl (CML model) AML model	*		1.8% of total diet energy	8 weeks	↓LSC population ↓Leukocytosis ↓Splenomegaly ↓Leukemia burden Via production of Δ12-PGJ3 Blocked by indomethacin and HQL-79	[52]
**U937-1**	Monocytic cell line	*		60, 120 and 240 μM	72 h	↓ Cell viability and proliferation (60 μM), ↑Apoptosis (120 and 240 μM), ↑Lipid droplet accumulation, ↑CD23 expression, Lipid peroxidation-independent mechanisms	[26]
**Raji, Ramos, U937-GTB, U937-1 and Mono Mac-6**	Highly sensitive	*	*	30, 60 and 120 μM	24 h and 72 h	↑ Apoptosis and necrosis (EPA) ↓ Cell multiplication ↑TAG-rich lipid droplets (EPA) ↑Lipid peroxidation Reversible by Vitamin E	[40, 71]
**Reh, Molt-4, Jurkat, K-562, and KG1-a**	Moderately sensitive	*	*	50 and 120 μM			
**KM-3, Nalm-6 and THP-1**	Slightly sensitive	*	*	120 μM			
**HL-60**		*		60 μM	72 h	↑Apoptosis (19%), ↑Necrosis (32%), ↓Proliferation, ↑Differentiation, ↑Lipid peroxidation, ↑Serglycin mRNA levels, ↑Production of superoxide anions, ↑G1 phase prolongation	[39]
**HL-60**	Human promyelocytic leukemia	*		10, 20, 50, and 100 μM	2, 4, 8, 12, and 24 h	↓Cell viability, ↑Apoptosis, ↑Necrosis, Induction of apoptosis by select dietary n-3 (EPA) and n-6 (GLA) polyunsaturated fatty acids	[28]
**ATCC, CRL-1923**	Chronic lymphocytic leukemia		*	10 μM	24 and 48 h	↓Cell viability, ↑Apoptosis, ↑Lipid peroxidation and generation of reactive oxygen species, ↓Activation of NF-κB, Exerts anti-inflammatory effects after linking to the G protein-coupled receptor 120 in macrophages	[29]
**Ramos and Jurkat cells**		*		30 μM	24 h	↑ Caspase 3 and 9 activities	[31]
**HL-60**	Human promyelocytic leukemia		*	0.4, 2, 5 and 10 μM	24 h	↓Sphingosine-induced apoptosis Inhibition of cytosolic phospholipase A2	[30]
**RBL2H3, HeLa**		*		25, 50, 75 and 100 μM	18 h	↑Apoptosis, ↑ Release of Cytochrome c, ↑Activation of caspase-3, ↑Hydroperoxide in mitochondria	[32]
**HL-60**	Human promyelocytic leukemia		*	50 μM	24 h	↑Apoptosis, ↑Activation of caspase-3, Bax-independent pathway	[34]
**RBL2H3**	Rat basophilic leukemia cells	*		50 μM	4 h	Increase calcium level, Generation of hydroperoxide, Induce apoptosis via Calcium-dependent hydroperoxide accumulation,	[33]
**HL-60**	Human promyelocytic leukemia		*	50 μM	24 h	↑Cell differentiation, ↑Growth inhibition, ↑NBT reducing activity, ↑Expression of c-jun mRNA, ↑ c-jun protein, ↓ Expression of c-myc oncogene	[35]
**RBL2H3**	Rat basophilic leukemia cells	*	*	25, 50, 75 and 100 μM	1 h	Suppress Th-2-seweked allergic immune responces, ↓Il-4 and IL-13 production, ↓ c-Fos expression, ↓NF-AT expression, ↓ Phosphorylation of extracellular signal-related kinase, ↓Phosphorylation of p38 mitogen-activated protein kinase by DHA only.	[44]
**Jurkat**	Human T cells		*	10 μM	3, 6, 12, and 24 h	↑Apoptosis, Cell cycle arrest ↑Ceramide levels → cdk2 inhibition → ↑PPa and PP2A activities → ↓Rb protein phosphorylation	[43]
**Jurkat**	Human T cells		*	60 and 90 μM	48 and 24 h, respectively	↑Apoptosis Caspase-3 cleavage, PARP degradation, and DNA fragmentation (via a phosphatase mediated process involving PP1 and PP2B)	[42]
**Jurkat**	Human T cells		*	15 μM	2 h	↓ viability, Caspase-3 activation, PP1-mediated	[7]
**HL-60**	Human promyelocytic leukemia	*		20, 35, and 70 μM	48 h	↓ proliferation, ↑Apoptosis Depletion of intracellular Ca^++^ → ER stress → UPR response (↑ protein levels of phosphorylated Eukaryote translation initiation factor 2a, a UPR hallmark)	[9]
**SP 2/0**	Mouse myeloma cells	*	*	5, 10, 20, and 40 μg/ml	24, 48, 72 h	↓ proliferation, ↓ Viability, Superoxides, prostaglandin and leukotriene-dependent	[27]
**T27A**	Mouse leukemia cell line		*	200-400 μM 1 mM	NM	Cytotoxicity ↑ tumor cell and lipid vesicle premeability	[86]
**HL-60**	Human promyelocytic leukemia	*		20, 40, 60, and 80 μM	24 h	↓ proliferation, ↑Apoptosis, Inhibition of topoisomerases I and II and DNA polymerases, Cell cycle arrest at G1/S phase, ↑Cyclin A and E protein levels	[87]

HSC, Hematopoetic Stem Cell; LSC, Leukemia Stem Cell; TAG, triacylglycerol; cdk2, cyclin-dependent kinase 2; Rb, retinoblastoma; NBT, Nitroblue Tetrazolium;

## *IN VITRO* STUDIES ON EPA AND DHA

### Anti-proliferative and differentiation inducing effects

Omega-3 FAs have been shown to have strong anti-proliferative and differentiation promoting effects [21, 22]. A natural lipid preparation rich in EPA and DHA (45.3% of the whole preparation) showed prominent anti-proliferative effects in promyelocytic leukemia (HL-60) cells. Separate treatment with pure EPA and DHA also inhibited the proliferation of these cells by 11.3 and 19.3%, respectively. However, these effects were more pronounced when they were used in combination with other fatty acids such as conjugated linoleic acid (CLA) [23]. DHA (10-50 μM), in combination with As_2_O_3_, induced cell death in HTLV-1-immortalized cells via decreasing mitochondrial membrane potential in a caspase- and PARP-1-independent pathway. This suggested a necrotic cytotoxic effect rather than apoptotic cell death [24]. A study on HL-60 leukemia cells showed that EPA (10 μM) is a potent inducer of ROS production and myeloid leukemia cell differentiation which gives it the ability to potentiate the differentiation-inducing effect of 12-O-tetradecanoylphorbol-13-acetate (TPA) [25]. The proliferation of monocytic U937-1 cells has been also shown to be inhibited by EPA at a minimal concentration of 60 μM which was accompanied with apoptosis induction at higher concentrations of 120 and 240 μM. However, in contrast to the results of a majority of studies, these effects were not attributable to the well-established peroxidation mechanisms since the addition of antioxidant agents did not interfere with the anti-proliferative effects of EPA [26]. A previous study on the mouse myeloma cells (SP 2/0) had also shown that EPA (5-40 μg/mL) and to a lower extent DHA exert anti-proliferative effects on this cell type which could be roughly imputed to their lipid peroxidation effects. Complete blockade of this effect by superoxide dismutase (SOD) and cyclooxygenase and lipooxygenase inhibitors revealed a role for superoxides as well as prostaglandins and leukotrienes [27]. A study on HL-60 leukemia cells evaluated the effects of EPA in combination with linolenic acid (GLA) on pulmonary inflammation. EPA (50-100 μM) noticeably increased apoptosis and reduced cell viability in HL-60 cells. This was associated with a significantly enhanced rate of necrosis [28]. DHA (10 μM) also induced apoptosis and reduced cell viability in the primary cells of CLL patients in a concentration- and time-dependent manner [29]. Conversely, in another study, DHA (10 μM) prevented sphingosine-induced apoptosis in HL-60 cells after 24 h [30]. Following EPA (30 μM) treatment, the cells showed increased caspase 3 and 9 activity without affecting caspase 8 activity or cell cycle progression [31]. Similar results have shown that EPA induces apoptosis via increased release of cytochrome c, activation of caspase-3 and enhanced mitochondrial-derived hydroperoxide (mitochondrial oxidative stress) [32]. To further investigate the mechanisms of EPA-induced apoptosis in mitochondria, RBL2H3 cells were treated with 50 μM EPA for 4 h. This significantly increased mitochondrial oxidative stress and calcium levels. The results showed that EPA can induce apoptosis via calcium-dependent hydroperoxide accumulation in the mitochondria [33].

Significant caspase-3 dependent apoptosis was observed following DHA (50 μM) treatment in HL-60 cells. The use of cyclosporine-A as a mitochondrial permeability transition pore (MPTP) inhibitor did not inhibit caspase-3 activation or Bax translocation indicating that DHA may induce apoptosis through a Bax-independent pathway [34]. Treatment of HL-60 cells with 50 μM DHA resulted in a concentration-dependent increase in cell differentiation and inhibited the cell growth. It also enhanced the nitro blue tetrazolium chloride (NBT) reducing activity as an indicator of cell differentiation. In addition, DHA (50 μM) noticeably increased c-jun mRNA and protein levels whilst those of c-myc were markedly reduced [35].

### Apoptotic effects

*In vitro* treatment of HL-60 and K-562 cells with EPA delayed cell cycle progression at G0/G1 but shortened the G2/M duration. It also induced significant necrosis in both cell lines by 19.6 and 4.4%, respectively. Moreover, apoptosis was induced by EPA to a much greater extent in HL-60 cells which was mediated mainly by downregulating the expression of the bcl-2 signalling molecule, a major regulator of apoptosis [36, 37]). Similar results were reported in another study indicating that EPA (40, 60, and 120 μM) can induce apoptosis in HL-60 cells but not in K562 cells independent of any of its effects on DNA fragmentation [38]. In addition, the apoptotic and necrotic effects of EPA have also been reported to be independent of both cyclooxygenase and lipooxygenase pathways and of lipid peroxidation [39]. Although the various EPA-sensitive and EPA-resistant leukemic cell lines express different levels of pro-apoptotic and anti-apoptotic factors such as c-myc and Bcl-2, the differences in the expression of these factors do not account for its differential effects on apoptosis of different cells [40].

DHA from sources other than fish oil such as *Crypthecodinium cohnii* algae also have notable cell growth inhibiting properties in HL-60 cells. Algae-derived DHA halted HL-60 cell proliferation by delaying the cells in G0/G1 in a concentration-dependent fashion (IC_50_ of 74 μM) and significant apoptosis was observed at 24, 48, and 72 h. This was associated with the inactivation of retinoblastoma (Rb) protein and up-regulating the expression of E2F-6, a suppressor of transcription, and the pro-apoptotic molecule Bax [41]. High concentrations of DHA (60 and 90 μM) induced Jurkat cell apoptosis via caspase 3 activation, PARP degradation and DNA fragmentation.

**Figure 2:**
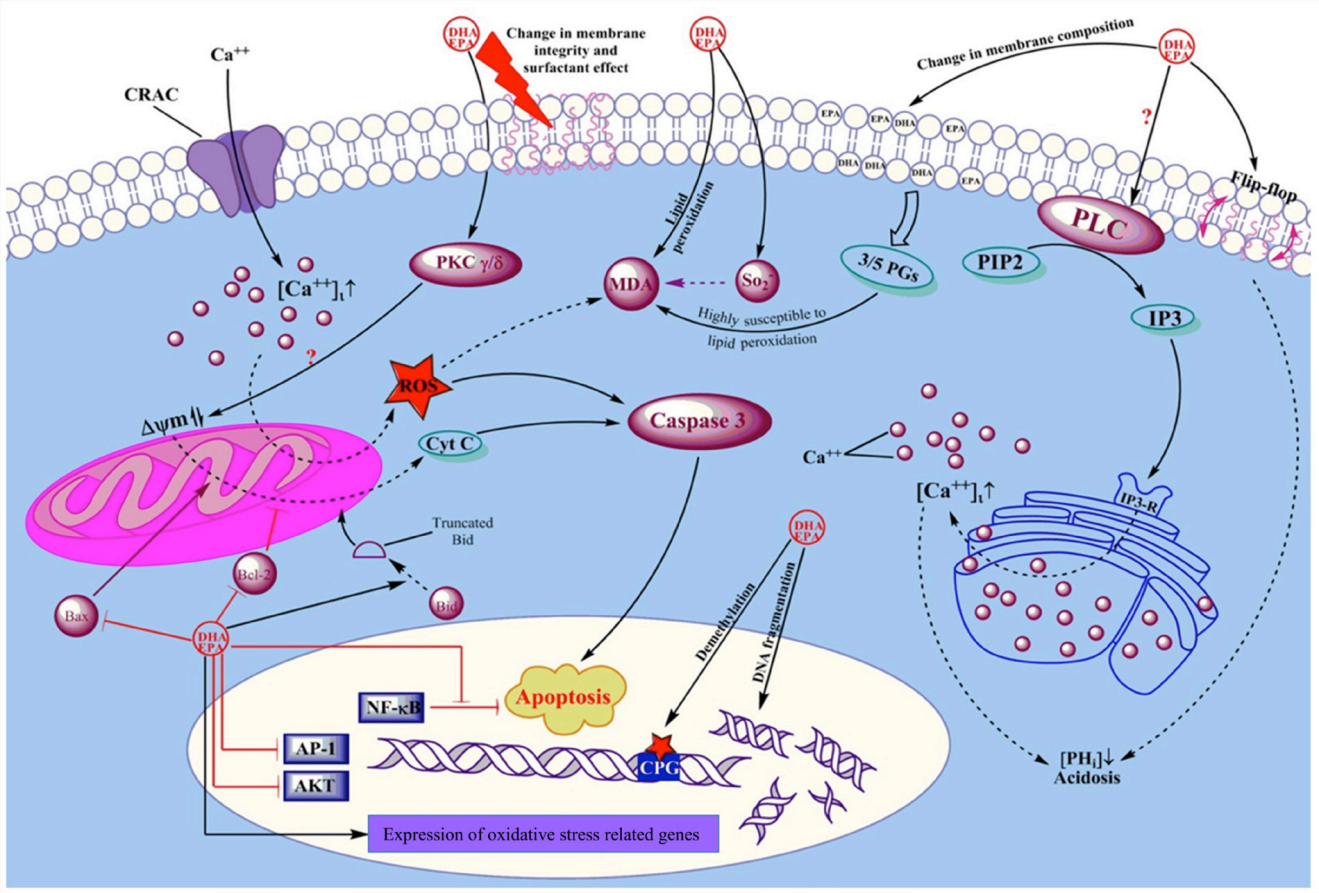
Schematic view of the mechanisms by which EPA and DHA affect leukemic cells Arrows show positive effect or activation. Dead-end lines indicate a negative effect or blockade. Question marks show controversial results from different studies. EPA and DHA exert their effects on cancer cells via changing the membrane compisition, altering intracellular Ca^++^ concentrations as well as intracellular PH, modifying mitochondrial membrane premeability, changing cellular resistance to ROS damage, and by direct actions on DNA and gene expression.

Tautomycin and cypermethrin, two phosphatase inhibitors which inhibit protein phosphatase 1 (PP1) and protein phosphatase 2B (PP2B) respectively, ablated the apoptotic effect of DHA implying a role for PP1 and PP2B in the induction of apoptosis [42]. The phosphatase-dependent cytotoxic effect of DHA (15 μM) has been also previously revealed in another study in which phosphatidic acid (PA), a specific PP1 inhibitor, reversed the DHA-induced apoptosis in Jurkat cells [7]. Repeated exposure to low concentrations of DHA (10 μM) rapidly decreased Jurkat cell viability whilst a single exposure at the same concentration only induced cell cycle arrest. Overall, DHA-induced Jurkat cell apoptosis and loss of proliferation are believed to result from increased ceramide production which leads to the decreased cyclin-dependent kinase 2 (cdk2) activity as well as increased PP1 and PP2A activities. This leads to the inhibition of Rb phosphorylation and subsequent cell growth arrest [43]. An overview of the effects of EPA and DHA in hematological cancers is illustrated in Figure 2.

Integration of EPA and/or DHA into the plasma membrane of cells decreases the proportion of arachidonic acid (AA) and consequently the production of AA-derived inflammatory mediators. In turn, the production of the less inflammatory EPA- and DHA-derived eicosanoids is increased. On the other hand, the ability of these two FAs to decrease the activity of regulatory T cells, major regulators of immune responses to leukemic cells, skews the immune response to act against hematological malignancies [11]. DHA and EPA can also prevent allergic diseases by inhibiting Th2-driven immune responses. The results showed that they mitigate IL-4 and IL-13 production, decrease expression of c-Fos and NF-AT whilst inhibiting phosphorylation of the extracellular signal-related kinase [44].

### Epigenetic alterations

DHA and EPA might also exert their biological effects via induction of epigenetic changes. These FAs are able to target the aberrant DNA methylation which is present in all types of cancers either across the whole DNA or at specific sites such as the non-coding regions of tumor suppressor genes [45]. Alteration of the extent of CpG methylation by PUFAs results in changes in the pattern of gene silencing and consequently the fate of target cell transcription. For example, EPA (100 μM) demethylates specific CpG sites within the promoter region of the tumor suppressor gene CCAAT/enhancer-binding protein (C/EBP)δ in the U937 promonocytic cell line leading to its enhanced expression. In addition, EPA and to lower extents other PUFAs such as DHA, increased the expression of transcription factors including C/EBPβ and C/EBPδ, PU.1 and c-Jun via similar epigenetic mechanisms [46]. Treatment of U937 cells with EPA (100 μM) can also activate the Ras/ERK/C/EBPβ pathway via demethylating a CpG island within an intron of the H-Ras gene which drives monocyte differentiation [18]. Overall, the anti-proliferative and differentiation promoting effects of EPA on monocytic leukemia cells is driven, at least in part, by demethylation of promoter regions of genes involved in tumor suppression and differentiation. These effects are not restricted to EPA as they also occur, albeit at lower potencies, with other PUFAs. EPA-induced down-regulation of serglycin mRNA is involved in HL-60 cell differentiation in a similar manner to that evoked by retinoic acid (RA). Nonetheless, this effect was not via actions on c-myc expression, a known mechanism of RA-induced differentiation [39]. In contrast, EPA treatment of U-937-1 cells does not increase the expression of the monocytic lineage differentiation markers, CD36 and CD68, at least at the concentrations tested [26].

### Effects on calcium homeostasis

DHA (10 and 20 μM) activates the PLC/IP_3_ pathway and thereby increases intracellular calcium (CA^++^) concentration and this has been implicated in DHA-induced apoptosis in U937 cells [47]. Ca^++^ mobilization by DHA also decreases intracellular pH which might play a role in DHA-mediated apoptosis and anti-proliferative effects in human T cells [48]. Econazole is an antifungal agent which mobilizes the endoplasmic reticulum (ER) Ca^++^ storage and blocks extracellular Ca^++^ influx leading to a constant depletion of ER Ca^++^ storage [49, 50]. In HL-60 cells, EPA induces apoptosis via the induction of ER stress causing the unfolded protein response (UPR) pathway to be activated. EPA triggers ER-stress via the depletion of intracellular Ca^++^ levels with a mechanism similar to that of econazole. In a similar fashion, EPA not only blocks trans-membrane Ca^++^ influx via store-operated Ca^++^ channels (SOC), but also it increases Ca^++^ release from the ER in an IP_3_-independent pathway. This will trigger the UPR response and finally lead to apoptosis [9].

## *IN VIVO* STUDIES

Dietary supplementation of 1.5% DHA (as an adjuvant to the nucleoside chemotherapy drug arabinosylcytosine, AraC) into a low-fat diet resulted in an average daily intake of 1.8 g DHA/kg^BW^/day and significantly increased the survival time of BDF1 mice bearing L1210 cell-induced leukemia from 12 days post leukemic cell injection to 33.3 days. In contrast, animals receiving higher amounts of DHA (4.5 g DHA/kg^BW^/day) survived for 26.5 days after leukemia implementation [51]. This indicates that there is an optimal dose of DHA required to enhance survival time. Dietary EPA supplementation in experimental CML and AML C57/BL6 mouse models has also been reported to target leukemia stem cells (LSCs) and decrease leukemia burden. Cyclooxygenase is necessary for the conversion of EPA into its metabolite Δ12-PGJ3. The inhibition of cyclooxygenase and hematopoietic prostaglandin D synthase (H-PGDS), by indomethacin and HQL-79 respectively, blocked the ability of EPA to attenuate cancer in CML-bearing animals. This suggests that Δ12-PGJ3 is the active agent for the EPA-induced effects which was confirmed with the re-establishment of the beneficial effects of EPA in indomethacin-treated mice following the administration of Δ12-PGJ3 [52].

### Effects of EPA/DHA on lymphoma cells

In a study by Speizer et al. which investigated the effects of fish oil FAs including EPA and DHA on the activity of protein kinases from lymphoma cells and bovine brain it was revealed that these FAs can differentially alter the activities of protein kinase C and cAMP-dependent protein kinase and thus are able to act as intracellular second messengers [53]. It is interesting to note that although EPA and DHA have been shown to enhance the number and function of B lymphocytes under normal conditions via altering the membrane composition of the cells, they have no such effects on Raji, Ramos, and RPMI lymphoma cell lines at least at the tested concentration (25 μM) and thus treatment with these two FAs does not promote cancer cell growth [54]. The suggested mechanism for this difference is the elongation of membrane-incorporated EPA and DHA into docosapentaenoic acid (DPA) which has been recently shown to substantially affect membrane properties [54]. EPA and more potently DHA were shown to readily incorporate into the plasma membrane of L5178Y lymphoma cells when added to the culture medium at concentrations higher than 10 μM. EPA and DHA also reduced the proliferation of the cells and increased the cytotoxicity of the chemotherapeutic agents including doxorubicin, dexamethasone, and mitomycin-C [55]. One study showed the necrosis and apoptosis inducing effects of EPA in two lymphoma cell lines, Raji and Ramos. The necrotic effect of EPA was reversed using different antioxidants without any effect on its apoptotic effects indicating a role for oxidative stress as a means of necrosis induction by EPA. In addition, protein synthesis was suggested to play roles in the EPA-induced apoptosis as this was prevented by the protein synthesis inhibitor cycloheximide [56]. Another study by the same group investigated the effects of EPA on the activities of different caspases and showed that the EPA-induced apoptosis in Ramos cells is mediated by a direct effect of EPA on the intrinsic pathway of apoptosis rather than interfering with cell cycle progression [57]. The use of triacsin C, an Acyl-CoA synthetase (ACS) inhibitor, has revealed the important role of ACS in the EPA-induced apoptosis. Inhibition of ACS by triacsin C caused a 90% decrease in the induction of apoptosis by EPA in the Ramos cell line. This can also, at least partly, explain the resistance of the U-698 cell line against EPA, since the U-698 cells express lower levels of ACS, compared to the EPA-sensitive cell lines such as Ramos [58].

A fish oil-supplemented diet has been shown to improve the metabolic parameters of dogs with lymphoblastic lymphoma. Such a diet resulted in increased plasma levels of DHA and EPA which was associated with lower levels of plasma lactic acid. This, in turn, increased the disease-free interval and survival time after doxorubicin chemotherapy [59]. The increased survival time and quality of life may not only have arisen from the direct anti-tumor activity of EPA and DHA, but also may be due to the cachexia-inhibiting effect of EPA, which was mediated by inhibition of cAMP in cancer cells both in mouse models and humans [60]. EPA, a key regulator of cyclin D1 synthesis, has been suggested to be of therapeutic potential in patients with mantle cell lymphoma since the main culprit in the pathogenesis and refractoriness of this type of malignancy is the elevated levels of cyclin D1 which is crucial in cell cycle progression from G1 to the S phase [61, 62]. This notion is further supported by the results of a study by Cvetkovic et al. which showed lower levels of PUFAs in the serum of patients with non-Hodgkin lymphoma in comparison with healthy controls and the association of lower amounts of PUFAs in the serum of these patients with the clinical severity and aggressiveness of the disease [63]. *In vitro* treatment of YAC-1 lymphoma cell line with EPA has been shown to induce apoptosis in these cells via a caspase-3 independent pathway. Increases in the cellular ROS production as well as the accumulation of lipid droplets containing triacylglycerol in the cells were among the important changes observed in the YAC-1 cells treated with EPA [64].

## DRUG SENSITIZING EFFECTS

Co-treatment of HL-60 leukemic cells with lipid preparations containing high concentrations of DHA and EPA did not improve the anti-proliferative effect of doxorubicin [23]. However, combining As_2_O_3_ and IFN-γ, a common chemotherapy for adult T-cell leukemia/lymphoma (ATL) patients, with emodin (a natural ROS-producing agent) and 10 μM DHA resulted in a 100-fold reduction in the dose of As_2_O_3_ required for the effective treatment of ATL. Emodin and DHA have been suggested to act via a synergistic action with A_2_O_3_ rather than having a direct anti-tumor action since treatment with emodin+DHA together did not induce significant effects on ATL. Potentiation of the cytostatic property of As_2_O_3_ by DHA was mainly attributed to its ROS generating abilities and down regulation of AP-1 and AKT expression which are both involved in cell survival [24]. As_2_O_3_, as an ROS-generating anti-cancer chemical, enhances ROS within malignant cells which, in turn, affects the co-administered DHA to form fatty acid hydroxides and hydroperoxides [65]. This potentiates the oxidizing actions of As_2_O_3_ which can be exploited to combat the malignant cells. In support of this, incorporation of 1 and 25 μM DHA into an As_2_O_3_-based regimen not only extended its apoptotic effect to the As_2_O_3_-resistant HL-60 cell line but also significantly decreased the viability of leukemic cells when compared to DHA treatment alone [14].

On the other side, there are various defence mechanisms by which cells protect themselves against oxidative stress. Glutathione peroxidase 4 (Gp-x4) is the most important and the only enzyme which neutralizes lipid hydroperoxide, an important DHA oxidation end product, which mediates the cytotoxic effects of DHA [66]. *In vivo* treatment of BDF1 leukemic mice with oral DHA increased the cytostatic effect of AraC but also abolished drug toxicity-induced death. Interestingly, this effect was reversed when the intake of DHA was increased from 1.8 g to 4.5 g/kg^BW^/day [51].

The biological effect of each member of PUFAs is specific to that member and differences between members of the same family of fatty acids have been reported. In this regard, treatment of HL-60 cells, bearing a Bcr-Abl translocation, with different concentrations of DHA (25, 50 and 100 μM) resulted in higher sensitivity of the cells to the tyrosine kinase inhibitor imatinib mesylate, increased percentage of apoptotic cells and reduced viability. In contrast, EPA, a member of the same FA family, had no effect at any of the concentrations tested. Loss of membrane integrity as well as increased DNA fragmentation was suggested to be involved in these effects [65]. Pre-incubation of the lymphoma cell line L5178Y with 10 μM and higher concentrations of DHA and EPA has been also shown to significantly increase the response of the cells to doxorubicin. The degree of unsaturation and chain length positively correlated with higher cytotoxic potencies of the tested FAs [55]. Cvetkovic et al. showed that the plasma phospholipid profile of patients with non-Hodgkin lymphoma influences response to chemotherapy in a manner that the plasma contents of PUFAs including EPA and DHA were lower in the patients who could not complete the chemotherapy course and in those with progressive disease compared to the patients in which the treatment period was successfully completed and remission was achieved [67].

Clioquinol, a recently used microbial agent in anti-tumor therapy, enhanced the apoptotic effect of DHA (100 μM) in a human B-cell lymphoblastoid line (Raji) after a 72 h combination therapy. Downregulation of NF-kB as well as reducing the levels of cell survival signalling molecules including Akt and Bcl-2 are involved in this synergistic effect. Although clinoquinol treatment alone did not induce lipid peroxidation, pre-treatment with vitamin E blocked the synergistic effect of DHA and clioquinol, indicating that lipid peroxidation might play a key role in DHA-induced toxicity in Raji cells [68].

Pre-treatment of B-CLL and B-PLL-derived cell lines (EHEB, MEC-2 and JVM-2, respectively) with different concentrations of EPA and DHA decreased the tolerance of the cells against doxorubicine-, fludarabine- and vincristine-induced toxicities resulting in decreased cell viability. The number of apoptotic cells also increased indicating that pre-treatment of the cells with DHA and EPA potentiated the cytotoxicity of the drugs via increasing lipid peroxidation, induction of ROS and inhibition of cell cycle at G0/M. Preventing oxidative stress using vitamin E did not reverse the drug-sensitizing effects suggesting the involvement of other unknown mechanisms [68]. DHA is also able to enhance doxorubicin sensitivity in P388 cells by elevating doxorubicin accumulation in cancer cells leading to increased cytotoxicity. A role for ROS was invoked as 50 μM DHA enhanced superoxide dismutase (SOD) and catalase (CAT) activities [69]. The drug-sensitizing effects of EPA and DHA have been summarized in Table 2.

**Table 2:** The drug sensitizing effects of EPA and DHA in combination with conventional chemotherapeutic drugs used for the treatment of haematological malignancies

DHA	EPA	Type of malignancy/model	Chemotherapy drug	Effect and suggested mechanism	Reference
*	*	Multiple myeloma cell lines	Bortezomib (Valcane)	NM	[20]
*		arsenic trioxide–resistant leukemic HL-60 cells (as well as solid tumor cell lines)	As_2_O_3_ [A ROS inducing chemotherapy agent]	↑Bax expression ↑Caspase 3 activation ↑ROS production and lipid peroxidation [Selective effect]	[13, 14]
*		HTLV-I–immortalized and –transformed T cells (a model of ATL)	As_2_O_3_	↑Cell death, ↓Proliferation, ↑ROS production ↓ mitochondrial membrane potential Down regulation of AP-1 and AKT expression [Selective; only marginal effect on healthy PBMCS]	[24]
*		BDF1 mice bearing L1210 leukemic cells	AraC	↑Survival time in ↓Drug-toxicity induced death	[51]
	*	HL-60 myeloid leukemia cell line	TPA	↑ROS production and NBT reduction ↑Differentiation of myeloid leukemia cells	[25]
*		Bcr-Abl expressing HL-60 cells	Imatinib	↓Viability, ↑Apoptosis, ↓Membrane integrity, ↑DNA fragmentation	[65]
*	*	B-CLL-derived cell lines EHEB and MEC-2 B-PLL-derived cell line JVM-2	Doxorubicin	↓Viability, ↑Apoptosis ↑ROS production ↑Lipid peroxidation	[85]
			Fludarabine		
			Vincristine		
*		T27A (murine leukemia cell line)	methotrexate	Growth inhibition via synergistic effect	

As_2_O_3_, Arsenic trioxide; ATL, Acute T-cell Leukemia; AraC, Arabinosylcytosine; TPA, 12-O-tetradecanoylphorbol-13-acetate; NM, not mentioned

## DISCUSSION

### Mechanisms of selective action

Although structurally similar, EPA and DHA can differentially affect the biological activity of cells, at least partly, due to their different magnitude of effects on AA-dependent processes within cells, as evidenced in U937 lymphoma cell line [70]. The long-term consumption of EPA has been shown to dramatically increase the EPA content of plasma, platelets and red blood cells with no effect on DHA content. This may suggests that extremely complex and distinct pathways may exist that regulate the metabolism of these two FAs [61]. Nonetheless, both EPA and DHA have selective actions on malignant cells with minor, if any, effects on normal cells [13, 14, 20, 24]. Therefore, the mechanisms responsible for these selective actions will not only help us design more effective anti-cancer treatments using omega-3 FAs but may also reveal new targets for chemotherapeutic agents. In contrast, cancer cell lines show variable levels of sensitivity to omega-3 FAs and some of them can tolerate treatment with these agents and are therefore resistant to them [40]. However, DHA and EPA can enhance the actions of other drugs to modulate the dysregulated activity/expression of oncogenic targets without having any significant effect on the target cells *per se*. An example of this is the ability of DHA and emodin to down-regulate the enhanced expression of AP-1 and increased AKT activity in HTLV-1 infected T cells [24]. Furthermore, since FAs synergize the oxidative effects of ROS-producing agents [13, 14] this may also elicit a potent anti-tumor action. However, the effectiveness of a single administration of DHA/EPA in the absence of ROS-producing agents indicates that this cannot be the only mechanism involved.

One of the mechanisms proposed for the anti-cancer effects of EPA and DHA is the induction of lipid peroxidation. The main intracellular mechanism which protects cells against lipid peroxidation is the enzyme GP-x4 and the variable sensitivity of different cell lines to DHA and EPA might reflect the differential expression of GP-x4 in various healthy and neoplastic cells. This will impact on the ability to protect against peroxidation-induced cellular damage [66].

The ability of different cells to metabolize EPA into its more effective metabolite Δ12-PGJ3 might also determine cell sensitivity to this FA [52]. Elevated expression of 3 acyl-CoA synthetases (ACSs) is observed in EPA-sensitive cell lines such as Ramos and Raji, giving these cells a greater ability to covert EPA into its active form EPA-CoA. In addition, some EPA-resistant cells such as U-698 have a limited ability to uptake PUFAs leading to reduced accumulation of cytosolic lipid droplets, a characteristic of responsive cells [26, 71]. The differential expression of apoptosis regulating genes such as c-myc which drives the cell towards a programmed cell death response in those which express higher levels of c-myc (Ramos cell line) or towards necrotic cell death in those with less c-myc may explain why some neoplastic cells treated with EPA/DHA undergo apoptosis and others undergo necrosis [40].

DHA alters the composition of membrane micro-domains replacing other fatty acids and reducing cholesterol and monoenoic FAs. This has been shown by evaluating the FA content of exfoliated microvesicles derived from DHA-treated leukemic cell lines. Thereby, DHA can induce a unique arrangement of lipid microdomains which might consequently determine the fate of membrane-bound signalling compartments to activate specific downstream pathways giving rise to different vital changes inside the cell [72].

### Feasibility of clinical usage

The significant anti-cancer properties of omega-3 FAs especially EPA and DHA as well as their selective action and high safety profile [73, 74] suggest a promising clinical future. To date, studies have investigated the correlation between fish oil, as a rich source of omega-3 FAs, and other seafood consumption with a reduced risk of cancers. However, few clinical trials have been conducted to specifically study the effects of EPA and DHA on hematological malignancies. A clinical trial on 12 ALL and 6 AML patients investigated the effects of EPA as part of an energy and protein dense supplement. At the end of the trial, alleviation of cancer-induced weight loss and improvement of the overall conditions of adult and pediatric patients was observed. Decrease in the levels of circulating cytokines and acute phase proteins were attributed to the desirable effects of EPA [75].

The results of phase I and II clinical trials in patients with solid tumors have demonstrated that omega-3 FAs are safe and show promising results especially in patients in which the blood concentration of these FAs was raised significantly after treatment [76]. The recommended dose of EPA to improve cancer-induced cachexia is 1.5-2 g/day and side effects at this dose are minimal [77]; however, the benefit of increasing doses is unclear [78, 79]. Although criticized by the fact that PUFAs increase oxidative stress and the production of lipid peroxyl radicals within cells predisposing them to oxidative damage [80], the consumption of PUFAs has been shown to be clinically safe. In one study, the safety of EPA oil was compared *in vivo* with the conventional commercial fish oil and the *in vitro* toxicity of EPA was assessed as well using the Ames mutagenicity test. The results showed that exposure of rats for 28 days at doses up to 2820 mg/kg/day does not cause any adverse effects on either of clinical, nutritional or anatomic pathology parameters of the animals. Moreover, EPA did not induce any mutations in the Ames test [81]. DHA is well-tolerated by humans even at doses up to 18 g/day [78]. In a study by Horrobin et al., the long-term intake of 12 g/day of ethyl-eicosapentaenoate as a dietary source of purified EPA by a patient suffering mantle cell lymphoma which resulted in a cumulative dose of 5 kg EPA within a 16-month period was shown to be safe without any side effects [61]. *In vitro* studies investigating the effects of DHA on different cancer cell lines have indicated IC_50_ concentrationsof 80-90 mM [66] which is within the plasma and tissue concentration ranges of EPA/DHA and can be achieved clinically. Enhanced efficacy may be obtained using albumin-bound EPA or DHA to negate the effects of protein binding [40]. Moreover, it has been shown that dietary EPA, when it is metabolized, can produce sufficient amounts of Δ^12^-PGJ_3_ to induce apoptosis in leukemia cells [82]. A very recent study which investigated the effects of dietary EPA and DHA supplementation on the nutritional inflammatory status and long-term survival of patients with acute and chronic leukemia and lymphomas (Hodgkin and Non-Hodgkin lymphomas) receiving chemotherapy revealed that the consumption 2 g/day of fish oil (containing EPA and DHA) for 9 months resulted in greater reduction in the C-reactive protein (CRP) and CRP/albumin ratio in the patients receiving the supplement and chemotherapy in comparison to those receiving chemotherapy only. This significantly prolonged the overall survival time of the patients [83]. The results of these studies all together may give rise to the notion that supplementation with PUFAs may not only cause direct anti-tumor effects in the context of haematological malignancies, but also it may improve the response to treatment and the side effects of conventional chemotherapy via attenuating the inflammatory status of the patients, thus playing a dual role in improving the treatment outcome of patients with haematological malignancies.

## CONCLUSION

Based on the previous findings, conducting studies comparing different cell lines in terms of resistance and sensitivity to EPA/DHA is recommended. This can help pointing out the differences which might serve as the target for the action of EPA/DHA. Moreover, considering the diverse effects observed with high and low concentrations of these two FAs, determining the optimal dietary/non-dietary supplementation of PUFAs into chemotherapy regimens is also essential. Delineating the precise mechanism of action of EPA and DHA in inducing selective cancer cell death; epigenetic effects on tumor suppressor genes, enhancing ROS production or effects on lipid peroxidation will enable better targeting of cancers using these drugs. While most *in vitro* studies conducted to date focus on the direct effects of PUFAs, *in vivo* studies utilising the synergistic actions of EPA and DHA may shed light on the indirect mechanisms of these agents such as on immune cell and cytokine regulation which might inhibit the growth of cancer cells. Rapid screening of primary cancer cells exposed to DHA and EPA ex vivo may also define the correct patient and combination of drugs required for optimal therapy enhancing our options for personalised or precision medicine.
